# The Sodium-Potassium Pump Controls the Intrinsic Firing of the Cerebellar Purkinje Neuron

**DOI:** 10.1371/journal.pone.0051169

**Published:** 2012-12-20

**Authors:** Michael D. Forrest, Mark J. Wall, Daniel A. Press, Jianfeng Feng

**Affiliations:** 1 Department of Computer Science, University of Warwick, Coventry, West Midlands, United Kingdom; 2 School of Life Sciences, University of Warwick, Coventry, West Midlands, United Kingdom; 3 Computational Systems Biology Centre, Fudan University, Shanghai, China; Georgia State University, United States of America

## Abstract

*In vitro*, cerebellar Purkinje cells can intrinsically fire action potentials in a repeating trimodal or bimodal pattern. The trimodal pattern consists of tonic spiking, bursting, and quiescence. The bimodal pattern consists of tonic spiking and quiescence. It is unclear how these firing patterns are generated and what determines which firing pattern is selected. We have constructed a realistic biophysical Purkinje cell model that can replicate these patterns. In this model, Na^+^/K^+^ pump activity sets the Purkinje cell's operating mode. From rat cerebellar slices we present Purkinje whole cell recordings in the presence of ouabain, which irreversibly blocks the Na^+^/K^+^ pump. The model can replicate these recordings. We propose that Na^+^/K^+^ pump activity controls the intrinsic firing mode of cerbellar Purkinje cells.

## Introduction

The cerebellum coordinates the execution and adaptation of motor behaviours [1]. Cerebellar architecture is based on repeats of a well defined connectivity motif, at the centre of which is the Purkinje cell. Physiologically, the intrinsic firing of Purkinje cells is modulated by synaptic inputs but to understand the cerebellar circuit it is important to first understand the properties of each component in isolation. In this study we investigate the isolated cerebellar Purkinje cell.

We propose that the Na^+^/K^+^ pump controls intrinsic Purkinje cell activity. The Na^+^/K^+^ pump uses the energy of one ATP molecule to exchange three intracellular Na^+^ ions for two extracellular K^+^ ions [2]. Thus the pump is electrogenic, extruding one net charge per cycle to hyperpolarize the membrane potential. *In vitro*, in the absence of synaptic input, Purkinje neurons can spontaneously fire action potentials in a repeating trimodal pattern that consists of tonic spiking, bursting and quiescence [3], [4], [5],[6]. The repeat length of the trimodal pattern is reportedly fixed for a single Purkinje cell but has been observed to vary among different Purkinje cells, in a range from 20 seconds to 20 minutes [3]. This second to minute duration of the trimodal behaviour is distinct from the millisecond timeframe of ion channel kinetics. However, changes in intra-and-extra-cellular ion concentrations can span seconds to minutes [7] and could drive the transition of modes in the trimodal pattern. We test this hypothesis with a realistic biophysical Purkinje cell model that can replicate the trimodal firing pattern. In this model, the Na^+^/K^+^ pump controls the transition from tonic to burst firing by regulating the extracellular K^+^ concentration ([K^+^]_o_), which rules a Kv1.2 K^+^ channel “gate” to bursting. And the Na^+^/K^+^ pump's electrogenicity generates the quiescent mode, upon regulation by the intracellular Na^+^ concentration ([Na^+^]_i_). During quiescence the ionic concentrations reset and the trimodal cycle starts again from the tonic phase. The Na^+^/K^+^ pump, as an enzyme, adheres to Michaelis-Menten kinetics [8] and its affinity for external K^+^ (K_K_) sets the duration of tonic firing and its affinity for internal Na^+^ (K_Na_) sets the total duration of firing (tonic+bursting). If these affinities are modulated to make the total firing duration shorter than the tonic, then the model cell fires in an experimentally observed bimodal pattern repeat of tonic spiking and quiescence [9]. If K_Na_ is set equal to (or lower than) the basal [Na^+^]_i_, then the model cell becomes locked in quiescence (a behaviour reported for Purkinje cells *in vitro*; [10]).

GABAergic stellate cells make inhibitory synaptic contacts upon the dendrites of Purkinje cells [11]. When these inputs are introduced upon the Purkinje cell model, with the Purkinje parameters set such that it fires in the trimodal pattern, they can switch it's firing from trimodal to bimodal. This aligns with *in vitro* experiments, where the firing of some Purkinje cells can be switched from an imposed bimodal pattern, to an intrinsic trimodal pattern, by pharmacological blocking of GABAergic synaptic inputs [3]. The firing of some other Purkinje cells is intrinsically bimodal and remains bimodal when synaptic inputs are blocked [9].

The Purkinje cell model can replicate the *in vitro* behaviour of the cerebellar Purkinje cell – it reconciles the divergent Purkinje behaviours observed in different *in vitro* studies and establishes a biophysical basis to this operating diversity (quiescent [10]; trimodal, [3]; bimodal, [9]). The activity of Purkinje cells recorded *in vitro*, with cerebellar slice recordings, might not correspond with the physiological activity of Purkinje cells *in vivo*. In the parasagittal cerebellar slice preparation, synaptic innervations to Purkinje cells are corrupted to a degree [3]. We discuss the physiological relevance of the model in the Discussion.

## Materials and Methods

### Ethics statement

Our experiments with rats were conducted in accordance with the UK Animals (Scientific Procedures) Act 1986 and associated guidelines. We had approval to conduct these experiments from the Biological Ethics Sub-Committee, of the Research Ethics Committee, at the University of Warwick. This committee scrutinizes in accordance with the appropriate guidance for such committees from the Home Office, the RSCPA and the Laboratory Animal Science Association (LASA). This committee ensures that staff and students are trained and experienced, and that the potential benefits of the research outweigh the effects on the animals concerned. The committee is committed to the promotion of the 3Rs (reduction, refinement and replacement). At the University of Warwick, highly qualified and experienced veterinary staff are actively involved in (1) the ethical scrutiny of research (2) the welfare, care and husbandry of animals and (3) provide advice and support to all staff and students involved in research using animals.

### Numerical simulations 1: Detailed Purkinje cell model

**Figure 1:**
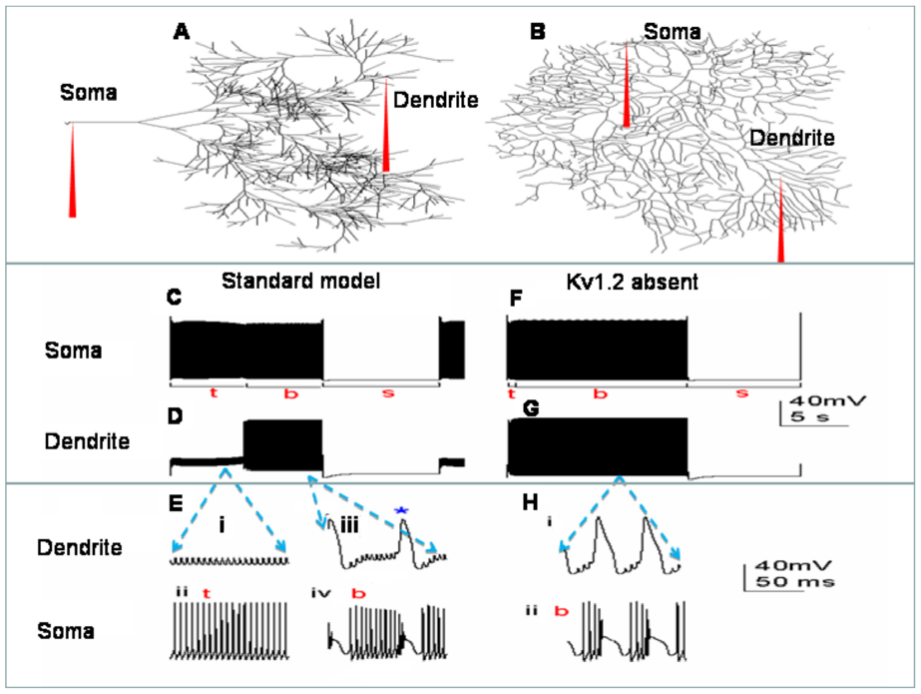
In the absence of synaptic input, the Purkinje cell model fires spontaneously in a repeating trimodal pattern. *A*, The model cell's default morphology with somatic and dendritic recording points labeled. *B*, An alternative morphology used for the model. *C*, A single trimodal repeat, recorded at the soma, with the constituent tonic (t), burst (b) and silent (s) modes labeled. *D*, The same trimodal repeat as (*C*) but recorded from a point within the dendritic tree. *E*, With no high-threshold spikes in the dendrites (*i*), firing is tonic at the soma (*ii*). With high-threshold dendritic spikes (*iii*), somatic firing has a stereotypical burst waveform (*iv*). A single dendritic spike is co-incidental with each somatic burst. The peak of the dendritic spike (* in sub-panel iii) causes the sudden and rapid depolarization that ends the somatic burst (*iv*). *F*, Single trimodal repeat recorded from the soma of a model variant that lacks the Kv1.2 channel. It has a markedly shorter tonic mode [t] than the standard model (*C*). *G*, The same trimodal repeat as (*D*) but recorded from a point within the model's dendritic tree. *H*, Without Kv1.2 channels, the dendritic spike frequency is higher (*i*) which results in a smaller number of spikes per somatic burst (*ii*). *Panels C, D, F and G are scaled by the first scale bar (40 mV, 5 s) and panels E and H by the second scale bar (40 mV, 50 ms)*.

Simulations were performed with the NEURON 5.6 simulator [12], using its backward Euler integration method and 25 µs time steps. The model's morphology is a detailed reconstruction of a Golgi-stained Purkinje cell (Fig. 1A), sourced from an adult rat [13] and discretized into 1089 compartments (soma: 1 compartment, smooth dendrites: 85 compartments, and spiny dendrites: 1003 compartments). Dendrite spines were not modeled explicitly; spines were accounted for by setting the spiny dendrites with a higher specific membrane capacitance [C_m_] (C_m_ = 0.8 µF/cm^2^ for the soma and smooth dendrites, C_m_ = 1.5 µF/cm^2^ for the spiny dendrites; [14]). Specific intracellular resistivity was set to 250 Ω/cm [14]. The model was also run with a different experimentally reconstructed Purkinje cell morphology from guinea pig (Fig. 1B) [15]. This morphology was discretized into 946 compartments (soma: 1 compartment, dendrites: 945 compartments) and no distinction was made in regard to smooth and spiny dendrites. Global specific membrane capacitance was 0.8 µF/cm^2^ and global specific intracellular resistivity was 200 Ω/cm [15].

The model incorporates twenty-one gated ion channels with descriptions established from the literature. The soma has highly TEA sensitive (I_K_fast_), moderately TEA sensitive (I_K_mid_) and TEA insensitive (I_K_slow_) voltage-gated K^+^ currents, a BK voltage-and-Ca^2+^-gated K^+^ current (I_BK_), a resurgent Na^+^ current (I_Na-R_), a P-type Ca^2+^ current (I_CaP_), a hyperpolarization activated cation current (I_H_), a leak current (I_L_) and an intracellular Ca^2+^ dynamics abstraction - all sourced from Khaliq et al. [16]. In addition, the soma has an SK Ca^2+^-gated K^+^ current (I_SK_) [17]. The dendrites have T-type (I_CaT_), Class-E (I_CaE_) and P-type (I_CaP_) voltage-gated Ca^2+^ currents; a leak current (I_L_); A-type (I_KA_), D-type (I_KD_), M-type (I_KM_), and delayed rectifier (I_DR_) voltage-gated K^+^ currents; BK (I_BK_) and K2 (I_K2_) type voltage-and-Ca^2+^-gated K^+^ currents and an intracellular Ca^2+^ dynamics abstraction - all sourced from Miyasho et al. [14]. In addition, the dendrites have a hyperpolarization activated cation current (I_H_) [18] and a Kv1 voltage-gated K^+^ current (I_Kv1_) [19]. The latter is incorporated as a Kv1.2 description because it replicates Kv1.2 kinetic data from a Purkinje neuron [9]. The model currents have equations and kinetic parameters as described in their source literature, but with the modification of current density values to those shown in Table 1. Current densities were assumed to be uniform over the dendrites (as in many other modeling studies e.g. [14], [20]) because there are not data to suggest otherwise and in this absence we were ruled by parsimony. This assumption makes for a smaller and more manageable number of model parameters.

**Table 1:** Maximal current conductances (mS/cm^2^) (assumed uniform over the dendrites).

Current	Soma	Dendrite
Resurgent Na^+^	156	0
P-type Ca^2+^	0.52	1.6
T-type Ca^2+^	0	0.6
E-type Ca^2+^	0	3.2
A-type K^+^	0	32
D-type K^+^	0	36
M-type K^+^	0	0.004
Delayed rectifier K^+^	0	0.24
Bk K^+^	72.8	60
SK K^+^	10	0
K2 K^+^	0	0.16
Kv1.2 K^+^	0	1
Highly TEA sensitive K^+^	41.6	0
Moderately TEA sensitive K^+^	20.8	0
TEA insenstitive K^+^	41.6	0
hyperpolarization activated cation, I_h_	1.04	0.29
Leak	0.1	0.08

The somatic Na^+^/K^+^ pump (density = d^s^_pump_, 1 mA/cm^2^) transports 3 Na^+^ out (i^s^_pump_Na_) for every 2 K^+^ in (i^s^_pump_K_) [2]. It has a fixed voltage dependency [21] and an exponential relation [22] to intracellular Na^+^ concentration ([Na^+^]_i_). The Na^+^ affinity constant (K_Na_) is 40 mM [2] and the cooperativity constant ([Na]_is_) is 1 mM [22]. *V* represents the membrane potential in mV as a dimensionless quantity.
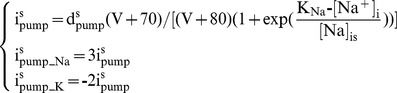
(1)The dendritic Na^+^/K^+^ pump (density = d*^d^*_pump_, 0.001 mA/cm^2^) has the same 3Na^+^:2K^+^ stoichiometry but no voltage dependency and a hyperbolic relation to extracellular K^+^ concentration ([K^+^]_o_) [23]. The K^+^ affinity constant (K_K_) is 2.245 mM [2].
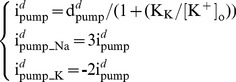
(2)Somatic intracellular Na^+^ concentration ([Na^+^]_i_) is initiated at 10 mM [22] and then changes in time *t* according to the relationship:
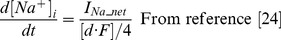
(3)

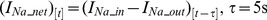
(4)

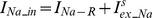
(5)

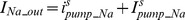
(6)Where i^s^_pump_Na_ and I^s^_pump_Na_ are Na^+^/K^+^ pump Na^+^ currents at the soma, set by eq. [1] and eq. [16] respectively. I_Na-R_ is the voltage-gated Resurgent Na^+^ current, I^s^_ex_Na_ is the Na^+^/Ca^2+^ exchanger Na^+^ current (set by eq. [15]), *F* is the Faraday constant and *d* is the somatic diameter. *I_Na_net_* (eq. [4]) is the difference between Na^+^ current flowing into the soma (*I_Na_in_*; eq. [5]) (Na^+^ ions flowing through the voltage-gated Resurgent Na^+^ conductance and the Na^+^/Ca^2+^ exchanger) and Na^+^ current pumped out of the soma by the Na^+^/K^+^ pump (*I_Na_out_*; eq. [6]), lagged by parameter τ = 5s. Tau (*τ*) is a factor that we have introduced and is not found in the original Canavier description [24]. Intracellular Na^+^ stimulates the Na^+^/K^+^ pump and this lag *τ* accounts for the duration of sodium's diffusion from channels to pumps. It aligns with the concept of a “fuzzy space” under the pump where the Na^+^ concentration differs from other parts of the cell [25]. The model represents Na^+^ diffusion abstractly, with this *τ* parameter, because a more explicit account would be ill constrained by the literature and too computationally expensive; intracellular diffusion processes have a much shorter spatial scale than electrical signalling and so their modeling requires a higher *nseg* value (the number of internal points at which NEURON computes solutions in each compartment; [12]) to attain spatial accuracy. The *τ* parameter is discussed much further in our Discussion section.

Extracellular K^+^ concentration ([K^+^]_o_) to the dendritic compartments is initiated at 2 mM and then changes in time *t* according to the relationship:
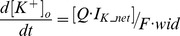
(7)

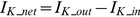
(8)

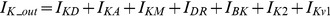
(9)

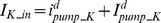
(10)Where *F* is the Faraday constant, *wid* is the thickness of an extracellular region around the compartment that K^+^ accumulates in (70*10^−3^ µm), Q is a K^+^ accumulation factor (0.143) and *I_K_net_* (eq. [8]) is the difference between K^+^ current flowing out of the compartment [*I_K_out_*] (through gated K^+^ conductances, eq. [9]) and K^+^ current pumped into the compartment [*I_K_in_*] (by the Na^+^/K^+^ pump, eq. [10]). K^+^ ions flow out of the model dendrites through the D-type (I_KD_), A-type (I_KA_), M-type (I_KM_), delayer rectifier (I_DR_), BK (I_BK_), K2 (I_K2_) and Kv1 (I_Kv1_) K^+^ currents. The Na^+^/K^+^ pump K^+^ current in the dendrites: i^d^_pump_K_ is set by eq. [2] and I^d^_pump_K_ is set by eq. [13].

A “ceiling” for extracellular K^+^ accumulation is set physiologically by the glial buffer system [26], [27] and accordingly the model [K^+^]_o_ is not permitted to exceed 3.03 mM [28] through code of the form: if ([K^+^]_o_>3.03) [[K^+^]_o_ = 3.03].

Formula [7] is a modification of the extracellular K^+^ accumulation equation employed by Durstewitz et al. [29] which is reproduced below: 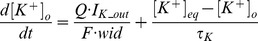
(11)Their *wid* setting is the same (70*10^−3^ µm) but Durstewitz et al. [29] utilise a *Q* value of 2 as opposed to our employed 0.143. We adjusted *Q* as a free parameter in our model tuning because this arbitrary factor is not constrained by the experimental literature. Durstewitz et al. [29] have no Na^+^/K^+^ pump mechanism in their model and hence no I_K_in_ parameter, only having an I_K_out_ parameter. Their formulation has an additional term on the right hand side (RHS), setting a decay to the extracellular K^+^ accumulation, where [K^+^]_eq_ is the equilibrium/resting value of [K^+^]_o_ and τ_K_ is the time constant with which it approaches this resting value. This term is an abstractive capture of cellular processes acting against extracellular K^+^ accumulation, primarily the action of the Na^+^/K^+^ pump (I_K_in_). In our work, we model the Na^+^/K^+^ pump explicitly and so this term is redundant and dropped from our description of extracellular K^+^ dynamics.

The model dendrites have two different Na^+^/K^+^ pump mechanisms. One has already been described (eq. [2]). The other is more abstractive (eq. [13]). It is included in the model to capture our hypothesis (which is founded in the experimental work of Genet and Kado, [30]) that the hyperpolarizing Na^+^/K^+^ pump current electrically balances a depolarizing Na^+^/Ca^2+^ exchange current. A simple Na^+^/Ca^2+^ exchanger mechanism is included in the model dendrites (eq. [12]). The use of an additional, simple Na^+^/K^+^ pump formalism, to offset the inclusion of a simple Na^+^/Ca^2+^ exchanger formalism, facilitated tuning the model such that the Na^+^/Ca^2+^ exchanger current was fully counter-balanced.

Convention permits inward (depolarizing) currents to be denoted negative and outward (repolarising) currents to be denoted positive [31]. The Na^+^/Ca^2+^ exchanger current (I^d^_ex_net_; eq. [12]) is *net* depolarizing (−1), inwardly passing 3 singly positive Na^+^ ions (3*[+1]) for the extrusion of every doubly positive Ca^2+^ ion (1*[+2]) [32]. By contrast, the Na^+^/K^+^ pump current (I^d^_pump_net_; eq. [13]) is *net* hyperpolarizing (+1) in its transport of 3 Na^+^ out (3*[+1]) for every 2 K^+^ in (2*[+1]).
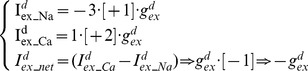
(12)

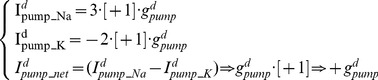
(13)g*^d^*_ex_ and g*^d^*_pump_ are Na^+^/Ca^2+^ exchanger and Na^+^/K^+^ pump membrane current densities (respectively) in the dendrites and their equality at 0.0021 mA/cm^2^ ensures an electrical counterbalance So, the model dendrites have a Na^+^/Ca^2+^ exchanger current and an electrically counterbalancing Na^+^/K^+^ pump current:
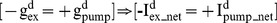
(14)The model's soma compartment also has a simple Na^+^/Ca^2+^ exchanger mechanism (eq. [15]; exchanger density = g*^s^*_ex_) and a counterbalancing, simple Na^+^/K^+^ pump mechanism (eq. [16]; pump density = g*^s^*_pump_). Thus, the soma compartment, like those of the dendrites, has both a detailed Na^+^/K^+^ pump description (eq. [1]) and a simpler Na^+^/K^+^ pump description (eq. [16]) in parallel.
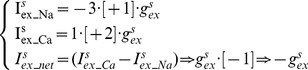
(15)

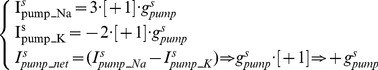
(16)In the soma compartment, the Na^+^/K^+^ pump current of equation [16] largely, but incompletely, counterbalances the Na^+^/Ca^2+^ exchanger current of equation [15] – there is a *slight* mismatch [g*^s^*_ex_ = 0.511 mA/cm^2^, g*^s^*_pump_ = 0.5 mA/cm^2^] (eq. [17]) which permits a small *net* influx of Na^+^ ions and a continued Na^+^ influx into the soma when the Resurgent Na^+^ conductance is removed to simulate TTX block of voltage-gated Na^+^ currents; this mismatch permits the model to replicate the Purkinje cell behaviour observed upon TTX application (refer Results).
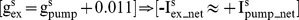
(17)The Purkinje cell model has four Na^+^/K^+^ pump equations ([1], [2], [13], [16]) and so four Na^+^/K^+^ pump densities (d^s^_pump_, d^d^_pump_, g^d^_pump_, g^s^_pump_) which we can represent as (d^x^_pump_, g^x^_pump_; x = s,d) where superscript [*s*] denotes a density at the soma and superscript [*d*] denotes a density in the dendrites. d_pump_ represents the density of a detailed Na^+^/K^+^ pump formalism (eq. [1], [2]) as opposed to g_pump_, which represents the density of a more simplified Na^+^/K^+^ pump description (eq. [13], [16]). So, the soma has a detailed and simplified Na^+^/K^+^ pump formalism (with densities [d^s^_pump_] and [g^s^_pump_] respectively) and the dendrites have a detailed and simplified Na^+^/K^+^ pump formalism (with densities [d^d^_pump_] and [g^d^_pump_] respectively).

The model's four different Na^+^/K^+^ pump equations are each valid and founded in previously published Na^+^/K^+^ pump descriptions. They capture different aspects of Na^+^/K^+^ pumping. The first captures [Na^+^]_i_ and voltage dependency (eq. [1]), the second captures [K^+^]_o_ dependency (eq. [2]), the third and fourth (eq. [13], [16]) are essentially equivalent and capture the electrical counterbalance to the Na^+^/Ca^2+^ exchanger current. It would be preferable to have these pump aspects in a single formalism. However, this would have greatly increased the complexity of tuning the model, which was methodologically unacceptable.

Ouabain irreversibly blocks the Na^+^/K^+^ pump [2]. An increasing proportion of Na^+^/K^+^ pump molecules being blocked by ouabain is replicated in the model by decreasing the model's four Na^+^/K^+^ pump densities (*d^x^_pump_*,. *g^x^_pump_*, x = s,d) by functions of time, *t* (in seconds): *Ouabain simulation is arbitrarily initiated at t = 3(s)*

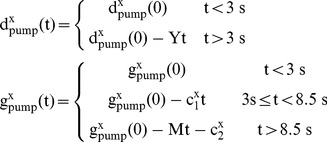
(18)Where x = s,d; Y = 0.1 mA cm^−2^ s^−1^; c_1_^s^ = 0.002 mA cm^−2^ s^−1^; c_1_^d^ = 0.01 mA cm^−2^ s^−1^; M = 0.1 mA cm^−2^ s^−1^; c_2_^s^ = 0.011 mA cm^−2^ s^−1^; c_2_^d^ = 0.055 mA cm^−2^ s^−1^

Catch coding, which is code of the form: if (x<0) [x = 0], is used to prevent negative values of (d^x^_pump_,. g^x^_pump_, x = s,d) from occurring.

To illustrate the sensitivity of the model to the K_Na_, K_K_ and τ parameters a variant of the model was constructed in which they were modified. K_Na_ was changed from 40 mM to 10.5 mM, K_K_ from 2.245 mM to 50 mM and *τ* from 5 s to 1 s.

GABAergic stellate inputs make inhibitory synaptic contacts upon the model dendrites; two inputs to every smooth dendrite compartment and one input to every spiny dendrite compartment [11]. They fire asynchronously, following a Poisson distribution around a mean frequency of input (1 Hz). Their reversal potential is −80 mV, with a synaptic weight of 0.001 µS and their amplitude upon activation follows a dual exponential time course (τ_1_ = 0.9 ms; τ_2_ = 26.5 ms) [11]. These model inputs can be removed to simulate pharmacological blocking of GABAergic synapses.

All parameters were established by prior literature (as referenced) except the twenty-one current densities, the Na^+^/K^+^ pump and Na^+^/Ca^2+^ exchanger densities, the synaptic weight, *Q*, τ and the parameters of Na^+^/K^+^ pump density decline used in the simulation of ouabain application (Y, c_1_^s^, c_1_^d^, M, c_2_^s^, c_2_^d^). These were all ill constrained by the literature and tuned manually [33] by iteratively running the model with different free parameter values and observing which combination of these gave the best fit between real and model Purkinje cell output. We tuned the model manually because, drawing from our experience, we reasoned that with the large number of parameters, and with the significant complexity/timeframe of the model behaviors sought, parameter optimization algorithms [34] would struggle to converge upon a good solution. Principally, we tuned the model to replicate the trimodal pattern of firing and when this behavior was captured, interestingly, the model could replicate a number of other Purkinje cell behaviors. For example, a P-type Ca^2+^ channel block switched the model from trimodal to bimodal firing, as has been observed experimentally (refer Results section).

### Numerical simulations 2: Reduced Purkinje cell model

To generate a simpler version of the described Purkinje cell model, the dendritic arbour was collapsed into fewer compartments with a reduction algorithm that conserves axial resistance (R_a_) [36], [37]. The method consists of merging successive dendritic branches into equivalent cylinders, preserving the axial resistance of the original branches. With this method, the radius (*R*) of an equivalent cylinder is given by:
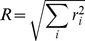
(19)Where *r_i_* are the radii of the collapsed branches for that equivalent cylinder. The length (*l*) of an equivalent cylinder is taken as an average of the lengths of the collapsed branches (*l*_i_) for that cylinder, weighted by their respective radii (*r*_i_):
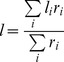
(20)With this collapsing method, the membrane surface area of the reduced model is less than the original because, although axial resistance is conserved, surface area (and hence the membrane resistance and capacitance) is not. This is compensated for by introducing, in each equivalent cylinder, a dendritic correction factor (*C*_d_), which rescales the current/pump/exchanger density values (*g*_i_) and membrane capacitance (*C*_m_) in the dendrites such that:
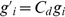
(21)

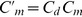
(22)The dendritic correction factor *C*_d_ is the ratio of the total surface area of the dendritic segments to their equivalent cylinders. The model's somatic compartment is not introduced into the collapsing algorithm and so in the reduced model it has the same dimensions as in the full model, and the *C*_d_ correction factor is not applied to any of its parameters.

Following the methodology of Destexhe et al. [37], our reduced Purkinje cell model had *C*_d_ applied to the model parameter, *depth*, which sets the depth of the sub-membrane shell that Ca^2+^ diffuses in within the model dendrites (the dendrite description of intracellular Ca^2+^ dynamics is sourced from Miyasho et al. [14]).
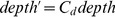
(23)There are a number of published mathematical transformations for collapsing dendrites into equivalent profiles (for a review, refer to [38]). We favored this “R_a_ conservation” algorithm because it has been shown to produce reduced, surrogate models that capture the synaptic integration properties of their full, parent models i.e. reduced models produced by this algorithm can perform the same non-linear integration of dendritic EPSPs and IPSPs and hence have the same input-output function as their parent models [36].

We used this “R_a_ conservation” algorithm to collapse the 1089 compartments of the full Purkinje cell model into the 41 compartments of a reduced model (1 soma, 40 dendritic compartments). The reduced model's 40 dendritic compartments were allocated smooth and spiny (20 smooth, 20 spiny) in an arbitrary mirroring of the full model (85 smooth, 1003 spiny). Note that in our approach, smooth and spiny dendrites are not distinguished from one another by the actual modeling of dendritic spines but by an abstraction, with a larger specific membrane capacitance (*C*_m_) for spiny dendrites (0.8 µF/cm^2^ for smooth, 1.5 µF/cm^2^ for spiny; [14]). *C*_d_ was 3.80. The dimensions of the soma were as in the full morphology (length = 22 µm; diameter = 22 µm) and the dimensions of the 40 dendritic compartments are shown in *Table 2*.

**Table 2:** Dimensions of the 40 dendritic compartments in the 41 compartment model.

Compartment	Length (µm)	Diameter (µm)
1	20.545455	3.3166248
2	19.4	2.236068
3	18	1.7320508
4	17.571429	2.6457513
5	8.5306122	3.3045423
6	13.344828	2.6305893
7	12.567568	2.8213472
8	16.9	3.1622777
9	11	3.3166248
10	10.352941	3.4467376
11	12.732394	3.9849718
12	12.5	3.7309516
13	10.475728	4.7791213
14	15.361446	4.3405069
15	11.986486	3.9899875
16	12.692308	5.5497748
17	10.326667	5.9665736
18	9.6402116	6.9079664
19	13.5625	6.2289646
20	9.8686831	6.5415792
21	10.347368	6.6932802
22	8.5744681	7.5232971
23	10.075188	8.2267855
24	9.5446429	7.3972968
25	8.6412429	9.1389277
26	8.8216783	8.5135187
27	8.1569732	9.4462488
28	6.1189159	8.5064444
29	7.8575581	10.055844
30	6.7650755	10.560032
31	6.892365	10.617743
32	6.5219251	11.041739
33	7.6343948	11.421714
34	7.9040284	10.478619
35	10.028048	10.878798
36	18.067147	9.2108216
37	17.821097	8.0038358
38	57.640576	7.3301444
39	24	3.5777088
40	18	4

Compartment 1 is linked to the somatic compartment.

The *Q* parameter is a setting in the model's description of extracellular K^+^ dynamics and in the full model it is 0.143. This parameter isn't set or constrained by any feature of the reduction algorithm used and is not constrained by any experimental literature. It is a free parameter. We manually tuned this parameter to confer a best fit between reduced and full model output, ultimately assigning it a value of 0.033. This is reasonably distinct from the value of 0.543 obtained by a *C*_d_ manipulation (0.143 * *C*_d_ = 0.543, where *C*_d_ is 3.80) but this is of no consequence, as there is no directive in the literature for applying *C*_d_ to such a parameter.

We strove for the smallest number of compartments, but through testing we found that 41 (1 soma, 40 dendrite) was the smallest number that could behave equivalently to the full 1089 compartments. This is because the collapsing algorithm does not conserve dendritic length fully, and Purkinje functioning requires a degree of uncoupling (distance) between somatic and dendritic events. 41 compartments is the smallest number that confers the sufficient distance (uncoupling) with a dendritic length of ∼502 µm. Without this sufficiency of length, for instance if the reduced model is the full model transposed by the algorithm to just 3 compartments (dendritic length of ∼129 µm), the tonic mode is absent in the trimodal firing pattern i.e. the model cannot replicate the Purkinje cell's trimodal firing pattern. By contrast, the reduced model of 41 compartments can replicate this activity motif.

We confirmed that the problem with the 3 compartment model was a lack of dendritic distance, rather than a lack of dendritic load, because increasing dendritic load could not enable a compartment number lower than 41. The “rallbranch” variable is unique to the NEURON simulator [12]. When a model compartment has a rallbranch number of *n* it is as though there are [*n*] mathematically identical compartments, including the entire sub-tree, connected to that compartment. This is a method of adding load to a dendritic tree without adding load to computation time. However, increasing rallbranch numbers could not decrease the compartment number requirement and thus it is not a load requirement that is limiting.

With another round of simplification, we produced a 5 compartment model that could reproduce the behavior of the full 1089 compartment model. Firstly, we collapsed the dendritic tree of the full 1089 compartment model to just 3 compartments (using the “R_a_ conservation” algorithm). We then overcome the dendritic distance issue by linking these 3 dendritic compartments to the somatic compartment through a coupling compartment that had a length of our setting, which we ensured was sufficient. This coupling compartment had the same passive and conductance properties as the dendritic compartments and, indeed, can just be thought of as component to the reduced model's dendritic tree. The coupling compartment and the next dendritic compartment along were set as smooth dendrites (*C*_m_ of 0.8 µF/cm^2^ to represent smooth; [14]) and the terminal two dendritic compartments were set as spiny (*C*_m_ of 1.5 µF/cm^2^ to represent spiny; [14]). We manually tuned the coupling compartment's dimensions to confer the right degree of uncoupling between soma and dendrites; to ensure that this 5 compartment model could replicate the behaviours of the 41 compartment and 1089 compartment models. In the tuned, and final, 5 compartment model: the total length of its dendritic compartments was ∼529 µm, its *C*_d_ value was 4.79 and [Q = 0.02] conferred a best fit to full model behavior. The dimensions of the soma were as in the full morphology (length = 22 µm; diameter = 22 µm) and the dimensions of the 4 dendritic compartments are shown in *Table 3*. The “coupling compartment” is compartment number 1 in this table.

**Table 3:** Dimensions of the 4 dendritic compartments in the 5 compartment model.

Compartment	Length (µm)	Diameter (µm)
1	400	3
2	16.11816	18.5088
3	95.16785	7.947963
4	18	4

Compartment 1 is linked to the somatic compartment.

Given that this 5 compartment model has some dimensions of our setting, its line of sight to the full model and the Biology is distorted. But at this cost it runs much faster, with 26 seconds of CPU time required for 1 second of simulation (Intel Pentium PC). This is ∼51* faster than the full model and ∼3* faster than the 41 compartment model.

In the Results section, all modeling results presented are from the full compartmental model (1089 compartments) unless specifically stated otherwise in the text.

### Preparation of cerebellar slices

Parasagittal slices of cerebellum (250 µm) were prepared from male Wistar rats, at postnatal days 28–35 (P28–35), with methods based on [10]. As described previously [35], and in accordance with the U.K. Animals (Scientific Procedures) Act (1986), male rats were killed by cervical dislocation and decapitated. The cerebellum was rapidly removed and slices were cut on a Microm HM 650V microslicer in cold (2–4°C) high Mg^2+^, low Ca^2+^ aCSF, composed of (mM): 127 NaCl, 1.9 KCl, 7 MgCl_2_, 0.5 CaCl_2_, 1.2 KH_2_PO_4_, 26 NaHCO_3_, 10 D-glucose (pH 7.4 when bubbled with 95% O2 and 5% CO_2_). Slices were stored in normal aCSF (1.3 mM MgCl_2_, 2.4 mM CaCl_2_) at room temperature for 1–6 hours before recording.

### Electrophysiological recording

Individual slices were viewed on a Zeiss FS Axioskop microscope with a 40× water immersion objective and Nomarski differential interference optics, at a total magnification of 640×. Slices were maintained at 30–32°C and continuously perfused (1–5 ml min^−1^) with a CSF, which was bubbled with 95% O_2_ and 5% CO_2_. Whole-cell patch-clamp recordings were made from visualized Purkinje cells using an EPC 8 amplifier (Heka, Digitimer, Welwyn Garden City, UK) controlled via a Digidata 1322a interface (Axon Instruments INC, Foster City CA, USA) using Clampex (v 9, Axon Instruments). Patch-pipettes (thick-walled borosilicate glass, Harvard Apparatus, Edenbridge, UK) were fire-polished, and had resistances of 1.5–4 MΩ when filled with an intracellular solution containing (mM): 135 K gluconate, 7 NaCl, 10 HEPES, 0.5 EGTA, 2 Na_2_-ATP 0.3 Na_2_-GTP and 10 mM Na phosphocreatine (adjusted to pH 7.2 with KOH and osmolarity adjusted to 300 mOSM with sucrose). Aliquots of intracellular solution were stored frozen at −20°C and thawed on the day of recording.

### Drugs

Drugs were dissolved at 1–100 mM in deionised water: bicuculline methiodide (Sigma), TTX (tetrodotoxin, Advent Scientific) and ouabain (Sigma). Aliquots of these stock solutions were stored frozen at −20°C, thawed and diluted in perfusion medium on the day of recording. All drugs were bath applied.

## Results

### The Purkinje cell model can replicate the trimodal pattern of spontaneous firing

In the absence of synaptic input, the model Purkinje cell fires spontaneously in a repeating trimodal pattern that consists of tonic spiking (*t*), bursting (*b*) and silence (*s*) (Fig. 1*C*). Significantly, the model captures the long timescale reported for Purkinje cell trimodality [3] with a pattern repeat length of ∼20 seconds.

There is some uncertainty as to whether the trimodal firing pattern is intrinsically generated or a function of neuromodulatory input [3]. This replication of trimodal firing in a realistic and experimentally constrained model of an isolated Purkinje cell adds to the case for intrinsic generation.

The model soma can fire Na^+^ spikes (Fig. 1*C*) and the model dendrites can fire Ca^2+^ spikes (Fig. 1*D*), in alignment with experimental findings [10], [39]. A comparison of Figure 1*C and 1D* shows that the model's tonic mode (*t*) is somatic spiking in the absence of dendritic spiking. Whilst the model's burst mode (*b*) is produced by dendritic spikes propagating to the soma to drive somatic bursting. Somatic bursts have a stereotypic waveform and each somatic burst is co-incidental with a dendritic spike (Fig. 1*E*).

The model's relationship between dendritic spikes and trimodal bursting is in alignment with experimental data, where a similar stereotypical burst waveform has been observed [5]. The frequency of dendritic spiking sets the model's burst parameters. For example, the Kv1.2 potassium channel limits the frequency of dendritic Ca^2+^ spikes and its removal from the model (Fig. 1, panels *F* and *G*) permits a higher frequency of dendritic firing (Fig. 1*H i*), which decreases the number of spikes per burst (Fig. 1H *ii*). This aligns with experimental data: pharmacological block of Kv1.2 channels increases the frequency of dendritic calcium spiking, and decreases the number of spikes per somatic burst, in the trimodal firing pattern of cerebellar Purkinje cells [9].

### In the model, the Kv1.2 channel gates the trimodal pattern's tonic to burst transition

The model dendrites have an intrinsic capacity to fire Ca^2+^ spikes. Their low-threshold T-type and Class-E voltage-gated Ca^2+^ channels open near the resting potential and they depolarize the membrane potential to activate high-threshold P-type voltage-gated Ca^2+^ channels. These P-type Ca^2+^ channels then produce Ca^2+^ spikes, which are repolarized by K^+^ flow through BK-type K^+^ channels (in alignment with Miyasho et al. [14]).

**Figure 2:**
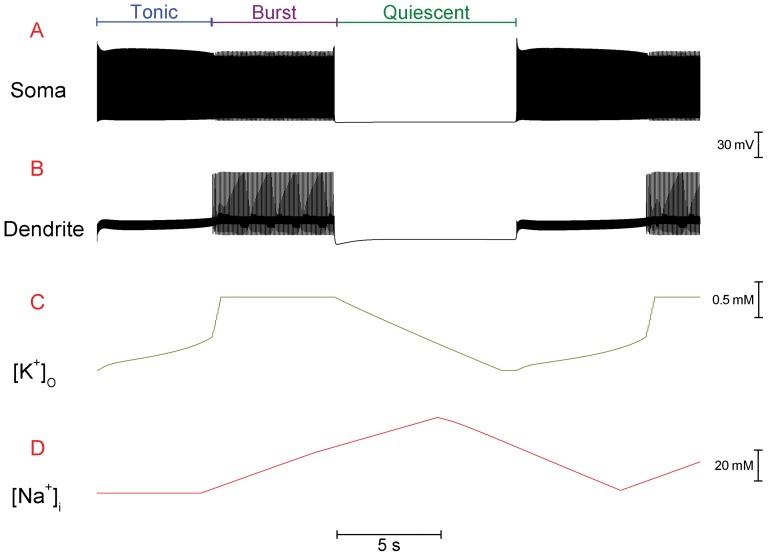
Changing ion concentrations drive the transition of modes in the Purkinje cell model's trimodal pattern of firing. All panels plot over the same period of time. *A*, Somatic membrane potential. The three different modes of a single trimodal repeat – tonic, burst, quiescent - are labelled. *B*, Membrane potential at a point in the dendrites. The burst mode at the soma corresponds with high threshold Ca^2+^ spiking in the dendrites. *C*, Extracellular K^+^ concentration ([K^+^]_o_) to the dendritic compartments. [K^+^]_o_ increases during firing as a function of voltage-gated K^+^ efflux. [K^+^]_o_ recedes (resets) during quiescence as voltage-gated K^+^ efflux stops whilst the Na^+^/K^+^ pump maintains K^+^ influx into the cell. [K^+^]_o_ cannot exceed 3.03 mM. This is our setting for the “ceiling” to [K^+^]_o_ accumulation (refer *Methods*) and is set for real Purkinje cells by the glial buffer system. As [K^+^]_o_ accumulation passes a critical point, firing is switched from the tonic to the burst mode. As described in the *Results*, this is because [K^+^]_o_ accumulation reduces the driving force, and current flow, for the Kv1.2 K^+^ current and this ultimately permits the dendrites to fire high threshold Ca^2+^ spikes which invade the soma and cause bursting. *D*, Somatic intracellular Na^+^ concentration, in a “fuzzy space” underneath Na^+^/K^+^ pumps ([Na^+^]_i_). [Na^+^]_i_ increases during firing, as a function of voltage-gated Na^+^ entry. However, changes in [Na^+^]_i_ are lagged by τ = 5 s to account for the duration of sodium's diffusion from channels to pumps (refer *Methods* and *Discussion*). As [Na^+^]_i_ increases past a critical point, the hyperpolarising force of Na^+^/K^+^ pumping achieves sufficient strength to drive the model cell to quiescence. [Na^+^]_i_ decreases during quiescence, as voltage-gated Na^+^ influx stops whilst the Na^+^/K^+^ pump maintains Na^+^ efflux. This decrease in [Na^+^]_i_ is lagged by τ = 5 s after the onset of quiescence.

This system is gated by dendritic Kv1.2 voltage-gated K^+^ channels. Kv1.2 channels generate hyperpolarizing current which clamps dendritic excitability and prevents Ca^2+^ spike generation, thus allowing the tonic mode of firing. However, the power of this excitability clamp diminishes with time because extracellular K^+^ accumulates (Fig. 2) and this reduces the electrochemical driving force for K^+^ flow. Eventually the hyperpolarizing current produced by Kv1.2 channel activity is insufficient to prevent dendritic spiking and the model is switched from the tonic to the burst mode.

Kv1.2 is low-voltage gated. In addition to Kv1.2, the model has other K^+^ currents/channels in its dendrites. However, these are largely uninvolved in the clamping of dendritic excitation as, unlike Kv1.2, they are high-voltage gated and not open at the relevant potentials. The D-type and A-type K^+^ currents are low-voltage gated but their involvement is limited because they inactivate quickly. By contrast, the Kv1.2 current is non-inactivating, which is why its current persists long enough to be tempered by slow ion relaxation processes. Indeed, experiment has shown the Kv1.2 current to be non-inactivating in the Purkinje cell (McKay, 2005).

The control of the tonic to burst transition by Kv1.2 channels enables the model to replicate an experimental investigation in which Kv1.2 channel block dramatically shortened the tonic phase within the trimodal pattern (Fig. 1*F*) [9].

### In the model, the Na^+^/K^+^ pump generates the trimodal pattern's quiescent phase

The Na^+^/K^+^ pump hyperpolarizes the membrane potential with a stoichiometry of three internal Na^+^ ions exchanged for every two external K^+^ ions (*methods*). The Na^+^ concentration in a “fuzzy space” underneath the pump ([Na^+^]_i_) rises during the tonic and burst firing modes (Fig. 2), as a function of voltage-gated Na^+^ entry, and this intracellular Na^+^ enzymatically increases pump activity (*Methods*). Eventually, during the bursting mode, the pump generates such a hyperpolarizing current that firing stops and the model cell is driven to quiescence. In this case, without any spike associated Na^+^ entry, [Na^+^]_i_ would be expected to stop rising. However, [Na^+^]_i_ continues to increase as the Na^+^ influx is lagged by a parameter *τ* (*refer Methods*), which phenomenologically encodes the long duration of Na^+^ diffusion from the Na^+^ channel to Na^+^/K^+^ pump (*refer Discussion for more detail and justification*). When *τ* expires, Na^+^ influx stops whilst the pump maintains Na^+^ efflux. This decreases [Na^+^]_i_, which reduces hyperpolarizing pump activity and eventually permits the model's spontaneous firing to resume. Firing resumes in the tonic spiking mode, rather than in the bursting mode which preceded the quiescence, as during the quiescence the Na^+^/K^+^ pump resets the extracellular K^+^ concentration and the Kv1.2 channels block to bursting. Thus, the trimodal pattern is reset for another cycle.

### The model can account for the heterogeneity in trimodal repeat length between different Purkinje cells

**Figure 3:**
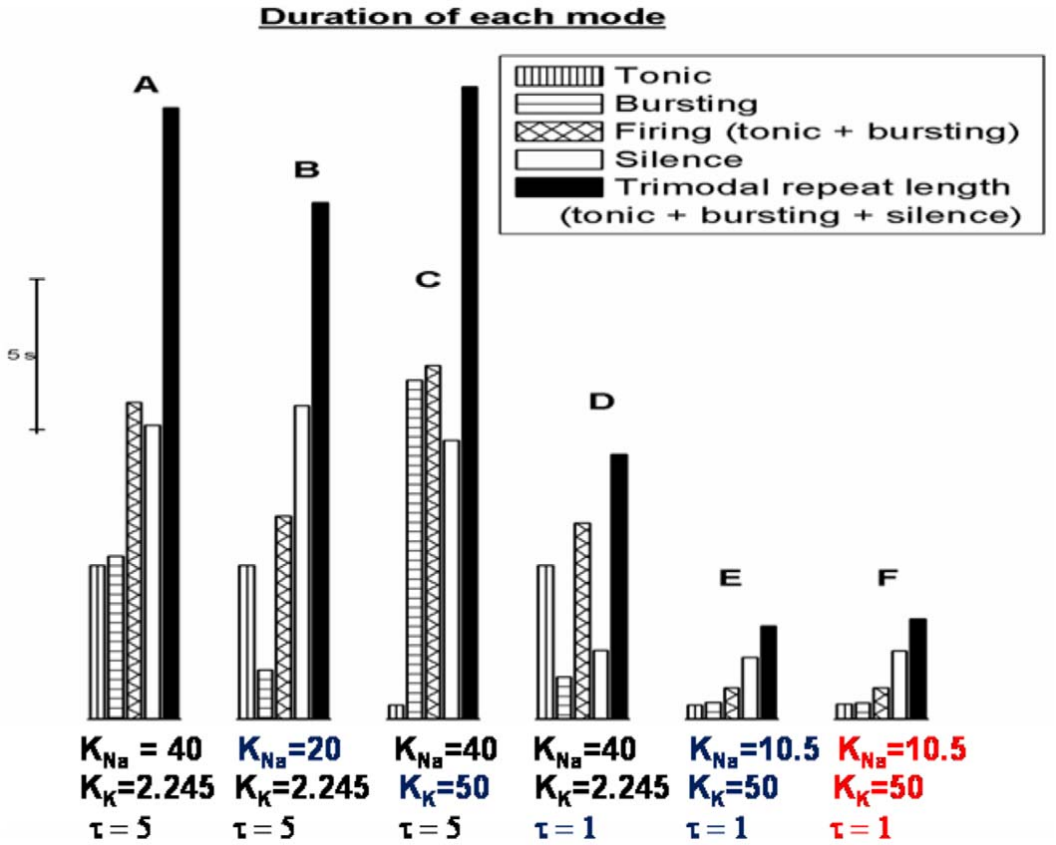
The K_Na_, K_K_ and *τ* model parameters set the model's trimodal repeat length. We present durations of the tonic mode, bursting mode, firing mode (tonic+bursting), quiescent mode and trimodal repeat length (tonic+bursting+quiescent) for different model settings. *A*, With default settings [K_Na_ = 40 mM; K_K_ = 2.245 mM; *τ* = 5 s] the trimodal repeat length is ∼20 s. *B*, Reducing K_Na_ (40 mM to 20 mM) shortens the firing (tonic+burst) duration. *C*, Increasing K_K_ (2.245 mM to 20 mM) shortens the tonic duration. *D*, Reducing *τ* (5 s to 1 s) shortens the quiescent mode. *E*, With K_Na_, K_K_ and *τ* all modified [K_Na_ = 10.5 mM; K_K_ = 50 mM; *τ* = 1 s] the trimodal repeat length is ∼3 s. *F*, When the model was run with a different reconstructed Purkinje cell morphology, but with the same parameters as (E), the trimodal repeat length was not majorly changed.

The trimodal repeat length is the duration of a single repeat of the trimodal pattern. It is described as a constant for an individual Purkinje cell but can reportedly vary from 20 seconds to 20 minutes between different cells [3]. The model replicates the fixed repeat length for a single cell (Fig. 3*A*) and provides a mechanistic basis to the cell-to-cell heterogeneity.

The Na^+^/K^+^ pump's affinity for internal Na^+^ (K_Na_ model parameter) sets the “firing length”, which is the combined duration of the tonic and bursting modes. The lower K_Na_, the smaller the quantity of intracellular Na^+^ that is needed to stimulate the Na^+^/K^+^ pump to a level of pumping that can hyperpolarize the cell to silence, which equates to a shorter period of Na^+^ entry i.e. a shorter “firing length” (Fig. 3*B*). Reported K_Na_ values vary in cardiac cells [2] and it is possible that K_Na_ could vary between cerebellar Purkinje cells (the Na^+^/K^+^ pump is present in both cardiac and neural cells. In the absence of relevant studies in neural cells, we cite studies in cardiac cells).

The Na^+^/K^+^ pump's affinity for external K^+^ (K_K_ model parameter) sets the duration of tonic firing. The higher K_K_, the shorter the tonic mode (Fig. 3*C*). K_K_ exerts this control by regulating the rate of extracellular potassium accumulation. Reported K_K_ values vary in cardiac cells [2] and it is possible that K_K_ could vary between cerebellar Purkinje cells.

Intracellular Na^+^ dynamics (encoded by the *τ* model parameter) set the duration of the quiescent mode. The smaller *τ*, the shorter the quiescent phase (Fig. 3*D*). Na^+^ dynamics are complex and embrace many constituent parameters, some of which could feasibly vary between different Purkinje cells.

K_Na_, K_K_ and *τ* are hypothesised to be fixed parameters for a single cell but with the potential to vary between different cells. A model variant was constructed in which these three parameters were modified. K_Na_ was changed from 40 mM to 10.5 mM, K_K_ from 2.245 mM to 50 mM and *τ* from 5 s to 1 s. The variant had a trimodal repeat length of ∼3 seconds (Fig. 3*E*) as compared to the ∼20 seconds of the standard model (Fig. 3*A*). This shorter repeat length has not been reported in the literature. The variant has the same model processes to generate its trimodal firing pattern, it is just that these processes run faster and take less time. This model variant is the standard model preparation for the remainder of this report as its shorter repeat length is more practical for the simulation of multiple trimodal repeats. Our Purkinje cell model is computationally expensive (22 minutes of CPU time required for 1 second of simulation; Intel Pentium PC) and using a briefer repeat length shortens the duration of simulations (of multiple repeats) to a manageable length. At the time of research, parallelization was not advisable for single neuron models in the NEURON environment because how to split the modeling of a single morphology among multiple CPUs was still an active research problem among NEURON developers. In any case, we did not have access to such a system. Biological modeling is typically abstractive, often because of computational constraints, and it is a judgment call where to apply this abstraction. We chose to have a realistic morphology and abstracted on the time course. The model is not a perfect simulacrum of a Purkinje cell and the conclusions we draw from it are tentative.

The model cell has a morphology reconstructed from a Purkinje cell in an adult rat cerebellum (Fig. 1*A*) [13]. When the model was re-run with the same parameters, but with a differently reconstructed morphology (Fig. 1*B*) (from guinea pig, [15]), the trimodal repeat length was not significantly changed (Fig. 3*F*). This is despite the second morphology being visually distinct. This suggests that morphology might not be a primary dictator of the trimodal repeat length. However, further work (with more Purkinje morphologies) is needed to draw a firm conclusion.

### The model's soma and dendrites can both fire spontaneously

**Figure 4:**
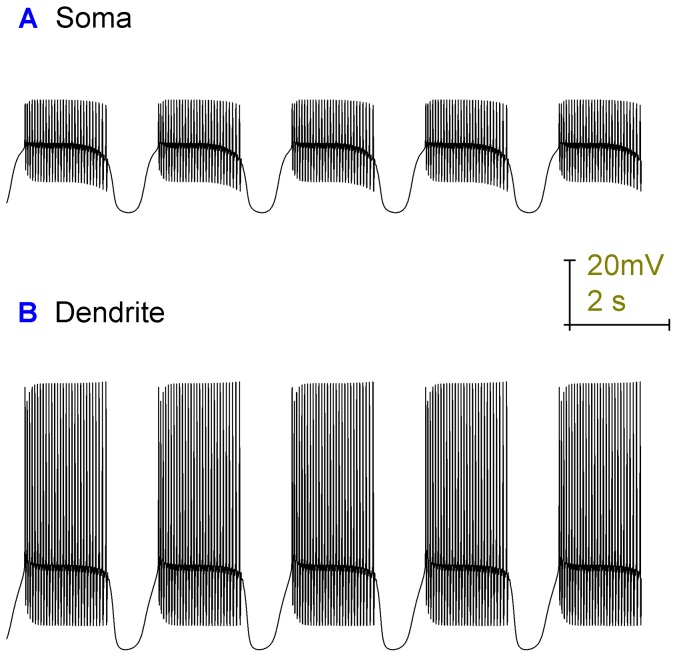
Removal of Na^+^ channels (simulated TTX block of Na^+^ channels) abolishes the model's Na^+^ spiking, at the soma, but not it's Ca^2+^ spiking in the dendrites. *A*, The model's somatic membrane potential (vs. Time), when Na^+^ channels are absent. There are no Na^+^ spikes but periods of small deflections in the membrane potential, which alternate with quiescent periods (bimodal behaviour). *B*, The model's dendritic membrane potential (vs. Time), when Na^+^ channels are absent. Ca^2+^ spikes persist in the dendrites and travel to the soma to produce the aforementioned small somatic deflections in membrane potential. This figure shows that the model's dendritic spiking is generated *de novo* and is not reliant upon an excitatory drive from the soma.

The model has a soma capable of spontaneously firing Na^+^ spikes and a dendritic tree capable of spontaneously firing Ca^2+^ spikes. Thus, the model's dendritic spiking is not reliant upon an excitatory drive from the soma. This enables the model to replicate the persistence of dendritic Ca^2+^ spikes when Na^+^ channels are blocked (by TTX; [5]) (TTX block is simulated in the model by setting the resurgent Na^+^ current density to 0) (Fig. 4). These dendritic spikes (Fig. 4*B*) propagate to the soma where they produce small deflections in the membrane potential (Fig. 4*A*). Hyperpolarization activated cation current (I_H_) is essential to the model's dendritic spontaneity. Without I_H_ in the dendrites, the dendrites cannot fire Ca^2+^ spikes spontaneously. They can only fire in the presence of somatic drive (data not shown). The importance of I_H_ in the Purkinje cell's electrical response to TTX has been shown experimentally [40].

In the presence of TTX, somatic activity is bimodal with periods of Ca^2+^ spike activity alternating with periods of quiescence [5]. The model can replicate this pattern (Fig. 4*A*), with the quiescent periods generated by the electrogenic action of the Na^+^/K^+^ pump (as with the trimodal pattern). In this TTX simulation, although there is no voltage-gated Na^+^ entry through the resurgent Na^+^ current, stimulatory Na^+^ to the Na^+^/K^+^ pump can still enter through the Na^+^/Ca^2+^ exchanger.

### The model can replicate other patterns of Purkinje cell firing

Some Purkinje cells express the trimodal firing pattern without pharmacological manipulation while others express it only upon the condition that GABAergic synaptic inputs are blocked [3]. In our experimental Purkinje cell recordings, we observed a repeating bimodal pattern of tonic spiking and quiescence when GABAergic connectivity is intact and only observed the trimodal firing pattern upon pharmacological block of GABA synapses.

**Figure 5:**
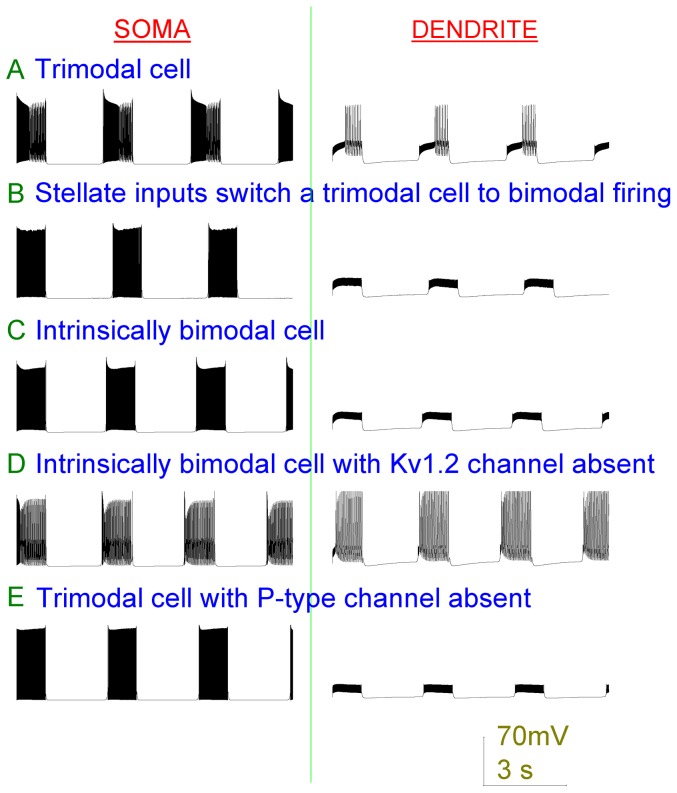
The Purkinje cell model can replicate experimentally observed bimodal patterns of Purkinje cell firing. Left panels are somatic membrane potential and right panels are dendritic membrane potential (vs. Time). By referring to the dendritic membrane potential one can distinguish bursting from tonic firing at the soma. Because bursting (unlike tonic firing) is co-incidental with dendritic spiking. *A*, The standard Purkinje cell model fires in a repeating trimodal pattern. *B*, Addition of inhibitory input switches the standard model out of the trimodal pattern and into a repeating bimodal pattern of tonic spiking and quiescence. *C*, Modifying the K_K_ parameter (50 to 0.8) makes the model intrinsically bimodal - firing bimodally (tonic spiking and quiescence) in the absence of inhibitory input. *D*, With the bimodal model of panel (C), removal of Kv1.2 channels switches it to fire in a trimodal pattern. *E*, With the trimodal model of panel (A), removal of P-type Ca^2+^ channels switches it to fire in a bimodal pattern.

GABAergic stellate cells make inhibitory synaptic contacts upon the dendrites of Purkinje cells [11]. When these inputs are introduced into the Purkinje cell model they clamp the dendritic excitability and prevent Ca^2+^ spike generation. Thus, they prevent the tonic to bursting transition and switch the model from firing in a trimodal pattern (Fig. 5*A*) to firing in a repeating bimodal pattern of tonic spiking and quiescence (Fig. 5*B*).

Some Purkinje cells intrinsically fire in this repeating bimodal pattern, in the absence of synaptic input [9], and with adjustment the model can replicate this behavior. For the model, we shall term the length of firing (tonic and/or bursting) that can happen before the pump gains enough strength to overcome it, and cause quiescence, as the “pump repeat length”. This is dictated by K_Na_. And we shall term the length of tonic spiking that can happen before the onset of bursting as the “tonic repeat length”. This is dictated by K_K_, which sets the loss rate of the Kv1.2 channel gate to bursting. These two lengths are fixed for a particular model cell but can vary between different model cells. If [pump repeat length>tonic repeat length] then the model cell fires in a trimodal pattern (Fig. 5*A*). If [tonic repeat length>pump repeat length] then the model cell fires in a bimodal pattern (Fig. 5*C*) - no bursting is observed because the pump drives the model cell to quiescence before it can occur. A model cell can be switched from bimodal to trimodal firing, or visa-versa, by changing the K_Na_ or K_K_ values to modify the relation of “pump repeat length” to “tonic repeat length”.

An intrinsically bimodal model cell can be switched to trimodal firing by blocking the Kv1.2 current (setting Kv1.2 channel density to 0) (Fig. 5*D*). This has experimental support with McKay et al. [9] observing that pharmacological block of Kv1 channels can switch a bimodal Purkinje cell into bursting; with the pertinent Kv1 channel reasoned by the authors to be Kv1.2. Physiologically, we can speculate that Kv1.2, with its acute phosphorylation control [41], [42], [43], might be an “online” molecular switch between the bimodal and trimodal firing modes.

A model cell firing in the trimodal pattern can be switched into the bimodal pattern by removing its dendritic P-type Ca^2+^ channel complement (Fig. 5*E*) such that it can no longer fire dendritic calcium spikes. This replicates Purkinje data recorded when the dendritic P-type Ca^2+^ channels were pharmacologically blocked [5].

Without synaptic input, not all Purkinje cells fire spontaneously - some are quiescent [10]. The model's basal intracellular Na^+^ concentration is 10 mM (*Methods*) and setting K_Na_ to 10 mM, which is a realistic value [2], locks the model cell in continuous quiescence (data not shown). It is worth mentioning that the persistently quiescent Purkinje state might be erroneous and that when quiescence is experimentally observed [10] it is not a continuous operating mode in its own right, but just a component of the trimodal or bimodal patterns. Our model is neutral to this debate, being able to replicate either scenario.

### The model recapitulates the response of Purkinje cells to Na^+^/K^+^ pump block by ouabain

**Figure 6:**
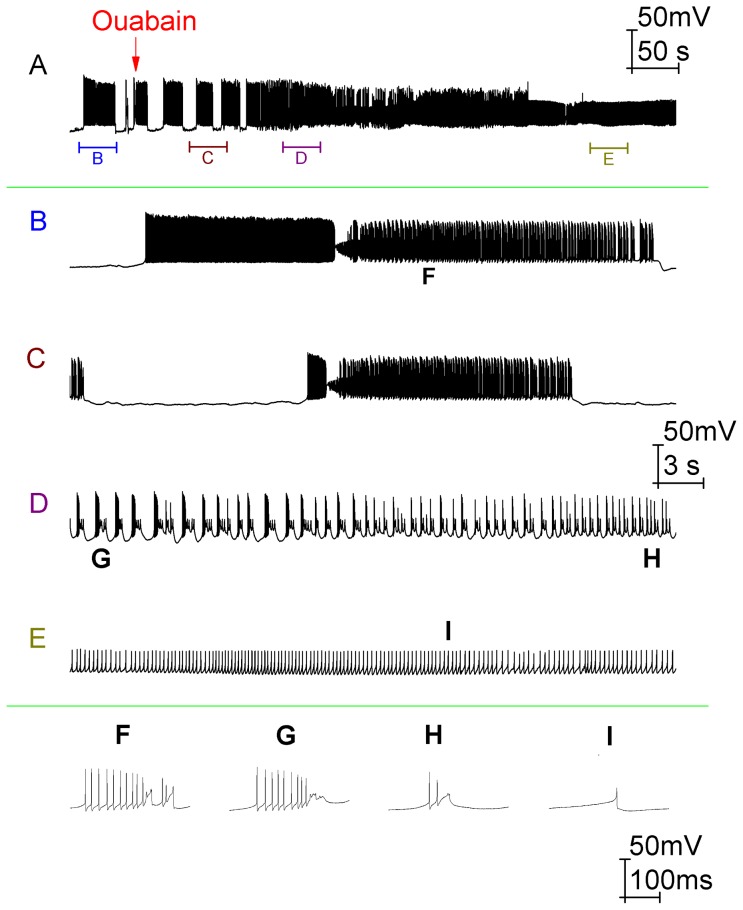
Ouabain block of the Na^+^/K^+^ pump switches Purkinje cell activity out of the trimodal firing pattern and into a continuous burst mode. Panel *A* shows a whole cell patch clamp recording from a Purkinje cell (with 10 µM bicuculline already present to block GABAergic inputs) following the application of 2.5 µM ouabain. The arrow denotes the time at which we feel that ouabain starts to modify Purkinje cell firing. Panels *B*, *C*, *D*, and *E* correspond to the labelled parts of panel *A*. Panels *F*, *G*, *H* and *I* show individual bursts from *B*, *D*, *D* and *E* respectively. The initial firing mode, before any Na^+^/K^+^ pump block, is trimodal (panel *B*). With time, as Na^+^/K^+^ pumps become blocked, the trimodal pattern's quiescent periods get shorter until they are eventually abolished and the cell fires continuously (panel *A*). Over this same period the length of the trimodal pattern's tonic mode also decreases, ceding to an increasing propensity to burst (compare panels *B* and *C*). Indeed, by the time of continuous firing (panel *D*) there is only bursting and no tonic fraction at all. After a period, this continuous bursting acquires a gradient of depolarization (Panel *A*). As it depolarizes, the number of spikes per burst steadily decreases (*F*, *G*, *H*) until the soma enters depolarization block and there are none. In this case, the only deflections observed are those of the Ca^2+^ spikes that have travelled into the soma from the dendritic arborisation (*E*, *I*). *Panel A scaling is encoded in the first scale bar (50 mV, 50 s). The scaling of panels B, C, D and E is encoded in the second scale bar (50 mV, 3 s). The scaling of panels F, G, H and I is encoded in the third scale bar (50 mV, 100 ms)*.

**Figure 7:**
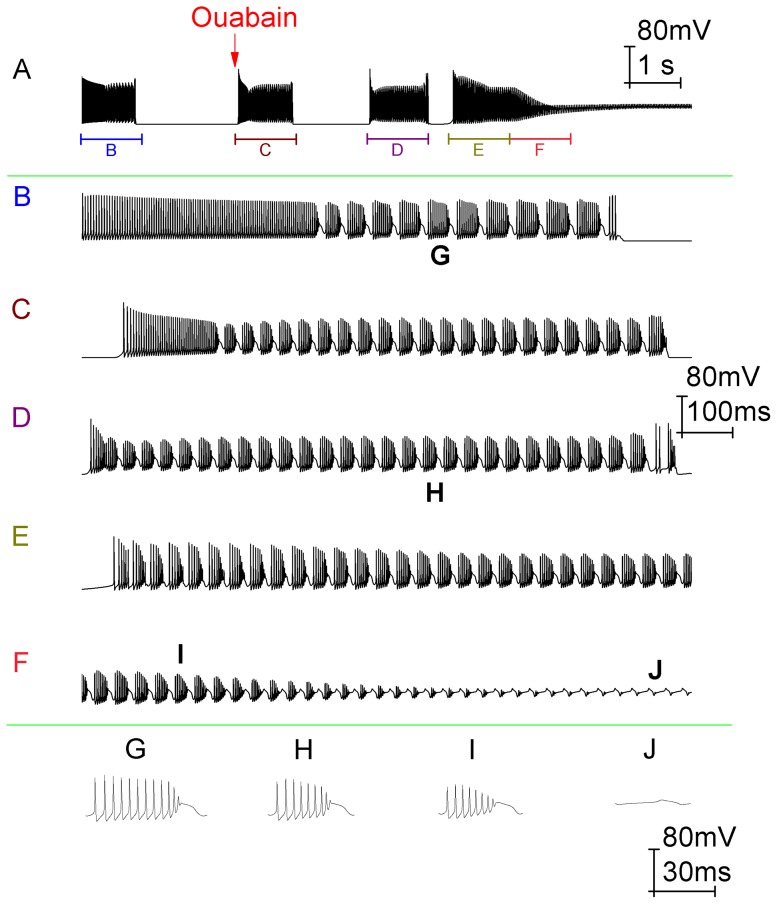
The model can recapitulate the response of Purkinje cells (firing in the trimodal pattern) to Na^+^/K^+^ pump block by ouabain. *A*, Somatic membrane potential (vs. Time) with the arrow denoting the onset of Na^+^/K^+^ pump block. Panels *B*, *C*, *D*, *E* and *F* correspond to the labelled parts of Panel *A*. Panels *G*, *H*, *I* and *J* show individual bursts from *B*, *D*, *F* and *F* respectively. The firing pattern in control, before any Na^+^/K^+^ pump block, is trimodal (panel *B*). Following the onset of Na^+^/K^+^ pump block, the trimodal pattern's quiescent periods get shorter until eventually they are abolished and the cell fires continuously (panel *A*). Over this same period the duration of the trimodal pattern's tonic mode also decreases, leading to an increased proportion of burst firing (compare panels *B*, *C* and *D*). Indeed, by the time cells are continuously active the firing only consists of bursts, with no tonic mode remaining (panel *E*). After a period, this continuous bursting acquires a gradient of depolarization (Panel *A*). As it depolarizes, the number of spikes per burst steadily decreases (*G*, *H*, *I*) until the soma enters depolarization block and there are none. In this case, the only deflections observed are those produced by Ca^2+^ spikes that have travelled to the soma from the dendritic arborisation (*F*, *J*). *Panel A scaling is encoded in the first scale bar (80 mV, 1 s). The scaling of panels B, C, D, E and F is encoded in the second scale bar (80 mV, 100 ms). The scaling of panels G, H, I and J is encoded in the third scale bar (80 mV, 30 ms)*.

As an objective assay of the model's validity we investigated whether it could predict the Purkinje cell response to the application of ouabain. This is an experimental test that the model has not been specifically tuned to capture and the ability of a model to predict/fit data not used in determining its parameters is an independent measure of how well the model approximates reality. Ouabain inhibits the Na^+^/K^+^ pump irreversibly [2] and thus, after ouabain application, the proportion of pump molecules blocked increases in time until eventually all pump activity is abolished. Ouabain application was simulated in the Purkinje cell model by decreasing the Na^+^/K^+^ pump densities by arbitrary functions of time (*Methods*), which switched the model's trimodal firing pattern into a continuous burst mode that converged upon depolarization block. This same transition was observed experimentally in whole cell patch clamp recordings from Purkinje cells (firing in the trimodal pattern) following the application of 2.5 µM ouabain (with 10 µM bicuculline present to block GABAergic inputs) (*n = 4*). After noting that the model and experimental response to ouabain matched, validating the model, we then tuned the model's pump density decline functions to generate a best fit between data (Fig. 6) and model (Fig. 7).

In the Purkinje cell response to ouabain (real or simulated), the duration of the trimodal pattern's silent periods became shorter until firing was continuous (Fig. 6*A*) (Fig. 7*A*). The duration of the trimodal pattern's tonic mode also decreased, leading to an increased proportion of burst firing (compare Fig. 6*B and 6C*) (compare Figs. 7*B, 7C and 7D*). Indeed, by the time cells were continuously active the firing only consisted of bursts, with no tonic mode remaining (Fig. 6*D*) (Fig. 7*E*). An experimental hyperpolarization of the membrane potential could not prompt the re-emergence of the tonic or quiescent modes (*n* = 3; data not shown). After a time, this continuous bursting attained a shallow gradient of depolarization that eventually converged upon somatic depolarization block (Fig. 6*A*) (Fig. 7*A*). This block was furrowed with small deflections in the membrane potential (Fig. 6*E*) (Fig. 7*F*), which are attributable to Ca^2+^ spikes that have travelled from the dendrites. So, although the soma was in depolarization block the dendrites were not and continued to fire. In some cells these deflections got smaller and smaller until there was complete quiescence, which suggests that in these cases the dendrites entered depolarization block as well (data not shown). During the progression to somatic depolarization block, the number of Na^+^ spikes per burst decreased (Figs. 6*F, 6G, 6H*) (Figs. 7*G, 7H, 7I*) until there were none and only Ca^2+^ spikes (from the dendritic arborisation) were observed (Fig. 6*I*) (Fig. 7*J*). During the somatic depolarization block, an experimental hyperpolarization of the membrane potential brought re-emergent Na^+^ spiking and showed this block to be a function of Na^+^ channel inactivation. However, this recovery could not occur if the hyperpolarization was introduced too long after the onset of depolarization block, perhaps because these Na^+^ channels become irreversibly inactivated in time (data not shown).

In this matching experimental and model response to Na^+^/K^+^ pump block, the loss of the quiescent mode indicates that the Na^+^/K^+^ pump generates trimodal quiescence. The loss of the tonic mode indicates that the Na^+^/K^+^ pump controls the tonic to burst transition through its setting of extracellular [K^+^], which controls a Kv1.2 channel “gate” to bursting. The depolarization block demonstrates that the Na^+^/K^+^ pump is required to counterbalance a strong and depolarizing force. Indeed, the pump has been reported to offset a depolarizing tetrodotoxin (TTX) resistant Na^+^ entry in the Purkinje cell [30]. The model incorporates our hypothesis that this Na^+^ entry is through the Na^+^/Ca^2+^ exchanger, which has been shown present in Purkinje cells [44]. The Na^+^/Ca^2+^ exchanger is *net* depolarizing (−1), inwardly passing 3 Na^+^ ions for the extrusion of every Ca^2+^ ion [32]. By contrast, the Na^+^/K^+^ pump is *net* hyperpolarizing (+1) with a stoichiometry of [3 Na^+^ out: 2 K^+^ in] [2]. The model soma and dendrites have a Na^+^/Ca^2+^ exchanger and an electrically counterbalancing Na^+^/K^+^ pump mechanism (*Methods*). With Na^+^/K^+^ pump loss the counterbalance to the depolarizing force of the Na^+^/Ca^2+^ exchanger is lost and depolarization ensues. The sizable Na^+^/Ca^2+^ exchanger density at the soma, when not counterbalanced by Na^+^/K^+^ pump, puts the soma into depolarization block. However, the lesser Na^+^/Ca^2+^ exchange density in the dendrites, when not counterbalanced by Na^+^/K^+^ pump, cannot put the dendrites into depolarization block.

Unfortunately, the ouabain response of the real and model Purkinje cell was not in complete alignment. The model's deflections in the membrane potential, furrowing the depolarization block, are smaller than those observed experimentally. Also, the model's timescale of response to ouabain (∼10 seconds, Fig. 7*A*) does not match that observed *in vitro* (∼1000 seconds, Fig. 6*A*). Although, to address the latter we propose that the model might be modified to operate over the longer experimental timescale by 1) changing K_Na_, K_K_ and *τ* to yield a longer trimodal repeat length and 2) using slower functions of decline for the model's Na^+^/K^+^ densities. To elaborate on point (2) - the effect of ouabain, which blocks a greater and greater fraction of Na^+^/K^+^ pumps over time, is replicated in the model by decreasing the model's Na^+^/K^+^ densities over time. The model parameters that set this rate of decline (Y, c_1_^s^, c_1_^d^, M, c_2_^s^, c_2_^d^) could be changed to pace the decline over ∼1000 seconds rather than ∼10 seconds – they are free parameters, unconstrained by any prior literature. However, the model takes 15 days to simulate ∼1000 seconds (22 minutes of CPU time required for 1 second of simulation, Intel Pentium PC) and it would take many trial and error runs (so many months, possibly years) to tune these free parameter values to give a best fit between model and reality. We believe that the model captures the processes that occur in real Purkinje cells under ouabain block, but that these processes run faster and take less time in the model i.e. the model abstracts on the time course. Biological modelling is typically abstractive, often because of computational constraints, and this can be permissible if it is clear where abstraction has been placed.

In experiments with a lower ouabain concentration (1.5 µM) the same sequence of events occurred but took longer (*n = 3; data not shown*), presumably because the pump block progression was slower. And with higher ouabain concentrations (10–20 µM), the progression to depolarization block was rapid and the transition through the aforementioned sequence was not observed (*n = 5; data not shown*).

**Figure 8:**
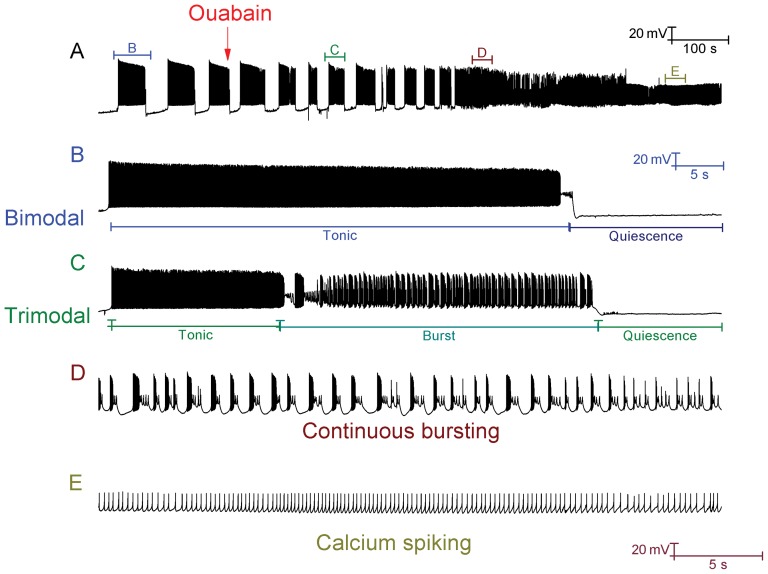
With inhibitory synaptic inputs unblocked, some Purkinje cells fire in a repeating bimodal pattern; ouabain block of Na^+^/K^+^ pumps switches these cells into the trimodal firing pattern and then a continuous burst mode. *A*, Whole cell patch clamp recording from a Purkinje cell (with GABA synaptic inputs intact/unblocked) in control and following the application of 2.5 µM ouabain. The red arrow denotes the time at which we feel that ouabain starts to modify Purkinje cell firing. Panels *B*, *C*, *D*, and *E* correspond to the labelled parts of panel *A*. In control, the Purkinje cell activity is in the repeating bimodal pattern (*B*). Following the addition of ouabain, the firing pattern is switched from bimodal to trimodal (*C*) and then transitions into continuous bursting (*D*). After a period, this bursting acquires a gradient of depolarization and ultimately converges upon a somatic depolarization block, in which the only deflections observed are those of Ca^2+^ spikes that have travelled into the soma from the dendritic arborisation (*E*). *Panel A scaling is encoded in the first scale bar (20 mV, 100 s). Panel B scaling is encoded in the second scale bar (20 mV, 5 s). The scaling of panels C, D and E is encoded in the third scale bar (20 mV, 5 s)*.

**Figure 9:**
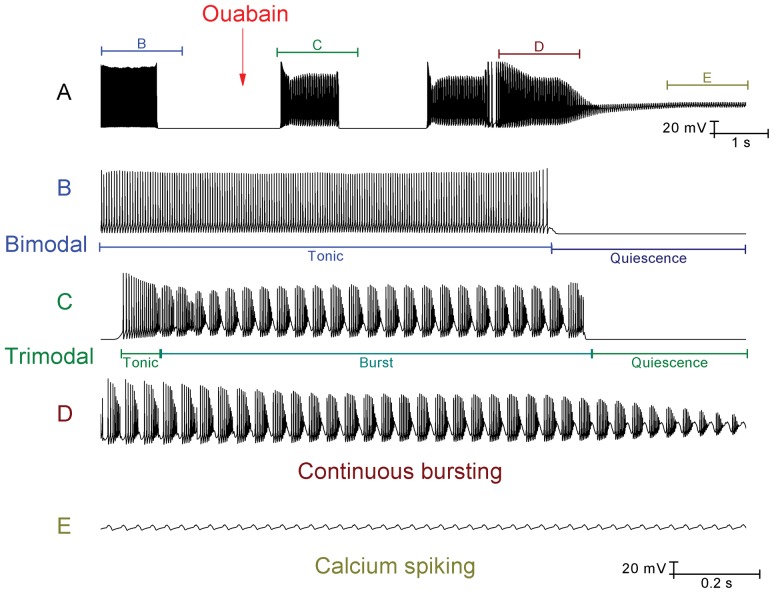
With inhibitory synaptic inputs unblocked, some Purkinje cells fire in a repeating bimodal pattern (tonic spiking/quiescence) and the model can replicate their response to Na^+^/K^+^ pump block by ouabain. Panels *B*, *C*, *D*, and *E* correspond to the labelled parts of panel *A*. *A*, With simulated GABAergic synaptic inputs innervating the model, it fires in a bimodal pattern of tonic spiking and quiescence (*B*). The red arrow denotes the onset of simulated ouabain application. Following this onset, the firing pattern is switched from bimodal to trimodal (*C*) and then transitions into continuous bursting (*D*). After a period, this bursting acquires a gradient of depolarization and ultimately converges upon a somatic depolarization block, in which the only deflections observed are those of Ca^2+^ spikes that have travelled into the soma from the dendritic arborisation (*E*). *Panel A scaling is encoded in the first scale bar (20 mV, 1 s). The scaling of panels B, C, D and E is encoded in the second scale bar (20 mV, 0.2 s)*.

Ouabain block of the Na^+^/K^+^ pump also imposes a switch in behaviour for Purkinje cells that have intact inhibitory synaptic inputs (no bicuculline applied) and which fire in a repeating bimodal pattern of tonic spiking and quiescence. Ouabain (2.5 µM) initially switches their firing from bimodal to trimodal, before a switch to continual bursting and an eventual depolarization block (Fig. 8) (*n = 3*). This indicates that the pump is the quiescence generating mechanism for the bimodal pattern of firing as well as for the trimodal pattern. The model is able to accurately reproduce this experimentally observed behaviour (Fig. *9*).

**Figure 10:**
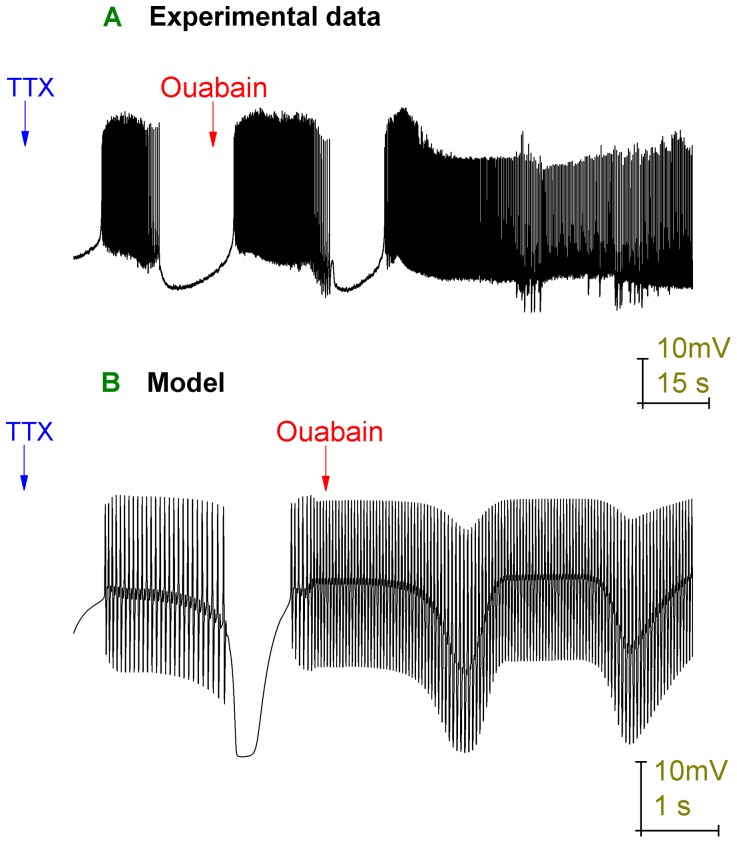
Upon Na^+^ channel block, real and model Purkinje cells express a repeating bimodal pattern (Ca^2+^ spike activity/quiescence) and Na^+^/K^+^ pump block eradicates its quiescent component. *A*, Whole cell patch clamp recording from a Purkinje cell in the presence of 1 µM TTX (to block Na^+^ channels), which sets a repeating bimodal pattern of Ca^2+^ spike activity and quiescence, before the introduction of 2 µM ouabain (to block the Na^+^/K^+^ pump) eradicates quiescence and renders continuous activity. *B*, The model's somatic membrane potential (vs. Time), with arrows denoting the onset of TTX simulation (Na^+^ channel density = 0) and ouabain simulation (Na^+^/K^+^ pump densities reduced as a function of time). The model replicates the principal features of the experimental recording. However, the model's transition into a continuous activity mode, without any quiescent periods, occurs faster than in experiment. Ouabain blocks a greater and greater fraction of Na^+^/K^+^ pump molecules over time, until all Na^+^/K^+^ pump molecules are blocked. Upon experimental ouabain application (panel *A*), one further quiescent period is observed before the rising ouabain block compromises a sufficient fraction, such that no further quiescent periods are observed. Upon simulated ouabain application (panel *B*), no further quiescent periods are observed. Ouabain application was simulated in the Purkinje cell model by decreasing the Na^+^/K^+^ pump densities by arbitrary functions of time (*Methods*). A slower transition, to the ouabain rendered behaviour, could be achieved in the model by using slower functions of decline for the model's Na^+^/K^+^ pump densities. However, the model does not employ such slower functions; it abstracts on the time course.

In the presence of TTX (1 µM), the Purkinje cell expresses a repeating bimodal pattern of Ca^2+^ spike activity and quiescence [5]. When Ouabain (2 µM) is applied to a Purkinje cell that is in this TTX induced bimodality, it shortens the length of the quiescent periods until ultimately the cell is switched to a pattern of continuous Ca^2+^ spike activity (Fig. 10*A*) (*n = 3*). This suggests that it is the Na^+^/K^+^ pump that generates the quiescent periods in the Purkinje response to TTX. This experiment is well recapitulated by the model (Fig. 10*B*).

### The reduced Purkinje cell models can replicate the behaviour of the full Purkinje cell model

**Figure 11:**
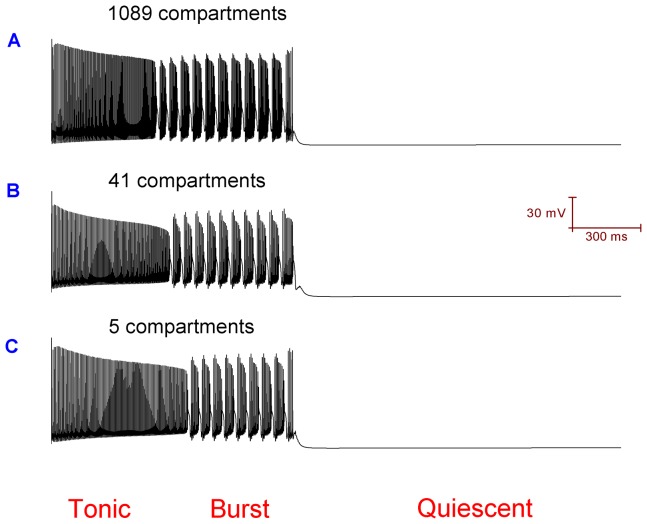
The 1089, 41 and 5 compartment models of the cerebellar Purkinje cell can all spontaneously fire in the trimodal pattern of activity.

**Figure 12:**
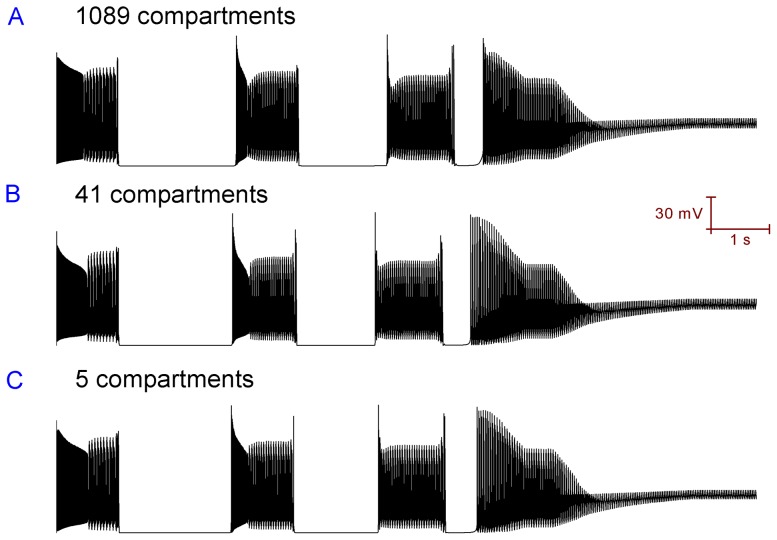
The 1089, 41 and 5 compartment models of the cerbellar Purkinje cell can all respond equivalently (qualitatively) to a simulated ouabain block of the Na^+^/K^+^ pump.

*Figure 11* shows that the 1089, 41 and 5 compartment models can all spontaneously fire in the trimodal pattern of activity i.e. they are qualitatively equivalent. *Figure 12* shows that the 1089, 41 and 5 compartment models all respond equivalently to simulated ouabain block of the Na^+^/K^+^ pump. This matching behavior might be an indication that morphology does not play a primary role in setting the parameters of the trimodal firing pattern (i.e. pattern repeat length, length of constituent modes).

## Discussion

### The Purkinje cell model can replicate the *in vitro* activity of the cerebellar Purkinje cell

This investigation valuably reconciles the divergent Purkinje behaviours observed in different *in vitro* studies (quiescent [10], trimodal [3], bimodal [9]) and establishes a biophysical basis to the operating diversity of the cerebellar Purkinje neuron. We suggest that at the foundation of the Purkinje cell's intrinsic multimodality, there is the working of just a single molecular species – the Na^+^/K^+^ pump - and we propose that it sets the Purkinje cell as persistently quiescent or spontaneously firing in trimodal or bimodal patterns (and sets the lengths of the different modes in these pattern repeats).

To date, the trimodal firing pattern has only been observed *in vitro* – it may be an *in vitro* artifact - and the length of its constituent modes (tonic, bursting, quiescent) is reported as fixed for a particular Purkinje cell [3]. However, if the trimodal pattern is physiologically relevant we propose that modulators to Na^+^/K^+^ pumping might act to regulate the length of modes as a signalling element to the deep cerebellar nuclei (DCN). The model shows how modifying the K_Na_ and K_K_ pump parameters, which might be modulated by signalling cascades *in vivo* [45], can change the trimodal pattern. Indeed, the model highlights that their modulation could be a molecular switch between the trimodal and bimodal patterns of firing.

The model shows that the trimodal pattern of firing is regulated by the extracellular K^+^ concentration. Neighboring Purkinje cells share an immediate extracellular milieu, which raises the possibility that adjacent or nearby Purkinje cells can signal to each other through the extracellular K^+^ concentration variable. This communication could be modulated by glial cells, which buffer K^+^ [46], and this would instill them as a novel working component of the cerebellar circuit.

### The *τ* parameter of the model captures the duration of Na^+^ diffusion from channels to pumps

The trimodal pattern's quiescent mode can span seconds or minutes [3] and thus its generative mechanism, which we suggest to be Na^+^/K^+^ pumping, must match this timescale. Na^+^/K^+^ pump activity is stimulated by intracellular Na^+^ and for it to persist at sufficient intensity during the quiescence, to perpetuate the quiescence, there must be a maintained intracellular Na^+^ accumulation. This persistence is non-intuitive as pump activity would be expected to quickly erode the Na^+^ accumulation during quiescence (by continuing to pump Na^+^ out of the cell in the absence of any voltage-gated Na^+^ entry), which would down-regulate pump activity. However, intracellular Na^+^ accumulation has actually been experimentally observed to persist minutes after the end of voltage-gated Na^+^ entry; a phenomenon that has been ascribed to a non-uniform cytoplasmic distribution of ions [47], [48], [49]. Neurons and muscle cells have a “fuzzy space” between the endoplasmic reticulum (ER) and the plasma membrane [25], [50], [51], [52], termed the “PLasmERosome”, where some “unknown molecular anatomy severely impedes the movement of small ions and yet allows equilibration with the cytoplasm over longer times” [53]. So, the soma has two spatially-distinct and weakly coupled compartments for Na^+^, the fuzzy space and the bulk cytoplasm. A steep Na^+^ gradient has been experimentally measured between these two spaces [47]. The Na^+^/K^+^ pump is located in the fuzzy space whilst the voltage-gated Na^+^ channels might be localised to a “third functional domain” [48] or they may also be in the fuzzy space but spatially segregated from the pumps, given this domain's reported microheterogeneity of high [Na^+^] “hot spots” alternating with areas of lower [Na^+^] [47]. Either way, the Na^+^ channel and pump appear geographically distinct. In the model, the lag parameter (*τ*) accounts for the duration of sodium's diffusion from channel to pump. This duration can be on the order of seconds to minutes because diffusion is significantly restricted in the fuzzy space by an, as yet, uncharacterized physiochemical process [54]. Thus, when Na^+^ influx stops it takes time to feed through and reduce the Na^+^ concentration underneath the pump, which is the critical concentration for pump activity. This lag could account for why the pump current decays on the “order of seconds to minutes”, as reported by Su et al. [55], rather than milliseconds. This large diffusive lag might also account for why no elevated pump activation is observed within 2 seconds of an increased Na^+^ entry [56]. The model represents diffusion abstractly, with the *τ* parameter, because an explicit account would be too computationally expensive and ill constrained by the literature. Intracellular Na^+^ and Ca^2+^ dynamics are linked (“cross-talk”) through the action of the Na^+^/Ca^2+^ exchanger and intracellular Ca^2+^ dynamics may be an additional complexity involved.

### Discussion of the Na^+^/K^+^ pump block experiments with ouabain

The reader must be aware that Na^+^/K^+^ pump block might eradicate quiescence in the trimodal and bimodal firing patterns, not because the Na^+^/K^+^ pump generates quiescence (per se), but because this block causes depolarization which compromises the activity of the true entity responsible. This is actually a problem inherent to many pharmacological block experiments that seek to parse the function of individual neuronal currents. By knocking out a current, the voltage trajectory is changed and in interpretation one is then unsure as to whether functional changes are due to that loss or due to changes in another current(s), which is regulated by the membrane potential. We did control experiments where we injected hyperpolarizing current to counter the ouabain induced depolarization (*n = 3*) and in which we still observed the loss of the quiescent mode (data not shown). However, this injection was at the soma and it probably didn't prevent depolarization in the entirety of the elaborate dendritic tree. So, it is with this caveat that we propose the Na^+^/K^+^ pump as the generative mechanism to trimodal quiescence.

Different cell types can have different response profiles to a Na^+^/K^+^ pump block. With hypoglossal motoneurons of the rat, “bath application of a 4–20 µM ouabain solution produced a partial block of Na^+^/K^+^ pump activity” and produced “no significant change in either the initial, early, or late phases of spike-frequency adaptation” i.e. 4–20 µM ouabain application did not cease or significantly alter the firing rate of rat motorneuons [57]. The authors of this study did “not believe that this negative result was a consequence of using ineffective concentrations of ouabain, because (they) often applied concentrations >10 µM which have been reported to inhibit ∼70% of the Na^+^/K^+^ activity in rat brain”. They reported that “ouabain concentrations >20 µM generally resulted in broad, short spikes and eventual cessation of repetitive discharge” (data not shown in their paper). By contrast, in our study with cerebellar Purkinje neurons of the rat, just a 1.5 µM ouabain solution can lead to the cessation of repetitive discharge. And this cessation occurs after a stereotypical sequence of changes, before any significant depolarization, that is not described in (and we presume is not applicable to) the motorneuron study of [57]: the quiescent and tonic periods of the trimodal firing pattern become shorter over time, whilst the burst phase of the trimodal firing pattern becomes proportionally longer.

Pharmacological block of the Na^+^/K^+^ pump has been shown to promote firing and prevent the generation of quiescent periods in canine coronary sinus fibers. This result is interpreted as evidence that the Na^+^/K^+^ pump is the generative mechanism to these quiescent periods [58]. This study is a precedent for our own Na^+^/K^+^ pump block experiments and our tentative conclusions from them. Although, incidentally, with coronary sinus fibers there is another layer of complexity that is not observed with cerebellar Purkinje cells - with Na^+^/K^+^ pumps blocked, quiescent periods can still occur in coronary sinus fibers, in some instances, all be it delayed. These are interpreted to occur because Na^+^/K^+^ pump block compromises the maintenance of the Na^+^ electrochemical gradient, which compromises Na^+^/Ca^2+^ exchanger activity, increasing [Ca^2+^]_i_ and raising Ca^2+^-activated K^+^ current flow, which hyperpolarizes the cell - overcomes firing - and generates quiescence. So, again, one can ascertain from this account that different cell types can have very different response profiles to a Na^+^/K^+^ pump block.

Before we considered the Na^+^/K^+^ pump as the drive to trimodal quiescence, we entertained the possibility that a Ca^2+^-activated K^+^ current might be responsible. However, we discounted the SK K^+^ current because the trimodal pattern's quiescent mode can still occur when this current is blocked by apamin [4]. And we discounted the BK K^+^ current because the trimodal pattern's quiescent mode can still occur when this current is pharmacologically blocked by IBTX [5] or when it is genetically knocked out [59]. In fact, the quiescent mode still occurs when BK and SK are blocked in concurrence by IBTX and apamin respectively [60]. And our Na^+^/K^+^ pump block experiment could indicate that Ca^2+^-activated K^+^ currents are not involved - Na^+^/K^+^ pump block has been shown to cause intracellular Ca^2+^ accumulation in a number of cell types because it compromises the Na^+^ electrochemical gradient, which the Na^+^/Ca^2+^ exchanger requires to pump Ca^2+^ out of the cell [58], [61]. So, if the trimodal pattern's quiescent mode is not generated by the Na^+^/K^+^ pump, but instead by a Ca^2+^-activated hyperpolarizing current, we might expect Na^+^/K^+^ pump block to promote quiescence rather than shorten and eradicate it. Indeed, as aforementioned, Na^+^/K^+^ pump block has been shown to cause quiescent periods via Ca^2+^-activated K^+^ currents in canine coronary sinus fibers [58].

### The model's mechanism of generating the trimodal pattern permits the model to replicate a diversity of other Purkinje cell activity states, which endorses this mechanism and the model

The detailed biophysical model replicates Purkinje cell firing patterns with the mechanisms that we hypothesise to be responsible in real Purkinje cells. And these predictions are forceful given the model's level of detail, with its faithfully reconstructed morphology and current, synapse and pump equations predominantly parameterized to experimental data. The model's mechanism of generating the trimodal firing pattern is endorsed by endowing the model with the ability to replicate a wealth of other experimentally observed Purkinje cell operating states: quiescence, the intrinsic bimodal firing pattern (Fig. 5*C*), the GABAergic imposed bimodal firing pattern that masks an intrinsic trimodal firing pattern (Fig. 5*B*), the stereotypical firing pattern observed in response to TTX block of Na^+^ channels (Fig. 4), Kv1.2 channel block switching Purkinje cells from the bimodal to trimodal firing pattern (Fig. 5*D*), P-type Ca^2+^ channel block switching Purkinje cells from the trimodal to bimodal firing pattern (Fig. 5*E*) and the complicated Purkinje cell response to an ouabain block of the Na^+^/K^+^ pump, both on its own (Fig. 7 and Fig. 9) and in combination with a TTX block of Na^+^ channels (Fig. 10).

The reduced models run faster than the full Purkinje cell model, yet faithfully reproduce its electrical behaviour. This similarity, between the outputs of the full model (1089 compartments) and its derivatives (the 41 compartment and 5 compartment models), serves to illustrate the reproducibility (semi-quantitative) of the computational experiments performed with the full model.

The mechanisms that we propose to drive the Purkinje cell's trimodal pattern of firing have been observed in other classes of neuron. Extracellular K^+^ has been experimentally observed to accumulate during sustained neural activity [26], [62], [63] and has been modelled to drive a tonic to burst transition in the neocortical pyramidal cell [7]. The electrogenic action of the Na^+^/K^+^ pump (regulated by the intracellular Na^+^ concentration) has been shown experimentally to generate quiescent periods in canine coronary sinus fibers [58].

### Physiological relevance of the model

*In vitro*, repetitive climbing fiber (CF) input (at a physiological frequency, ∼1 Hz) switches Purkinje cells out of the trimodal firing pattern and into a nonbursting pattern of activity [63]. On this basis it is probable that the trimodal firing pattern is not applicable *in vivo*. However, in these experiments, although CF input blocks the trimodal pattern's bursting mode, quiescent periods can still be observed.

CF input has been observed to toggle the cell between a tonic firing state and a quiescent state. So, state transitions occur at the frequency of CF input (∼1 Hz) and tonic firing periods of ∼1 s alternate with quiescent periods of ∼1 s (*in vitro* [64]; *in vivo* [65]). We do not propose that the Na^+^/K^+^ pump generates these quiescent periods. However, there are much longer quiescent periods observed, where CF input cannot evoke a complex spike or a state transition to tonic firing (Fig. 1*D* in [64]), and we propose that these are Na^+^/K^+^ pump generated silences. Indeed, a sizable fraction of the quiescent periods observed *in vivo* have an onset and termination that is not co-incidental or driven by complex spike events [64]. So, we speculate that although CF input blocks the trimodal pattern's bursting mode, it does not block its quiescent mode. Which we have shown could be generated by Na^+^/K^+^ pumping i.e. we tentatively suggest that Na^+^/K^+^ pump generated silences occur physiologically and speculate that they have a function. Interestingly, mutations in the Na^+^/K^+^ pump can cause rapid-onset dystonia-parkinsonism (RDP), which has symptoms to indicate that it is a pathology of cerebellar computation [66], [67]. In addition, there is a growing body of work indicating that Na^+^/K^+^ pumps might subserve information processing roles in neurons [68], [69], [70], [71].
